# Characteristics of wood apple (*Limonia acidissima* L.) and soybean powder jelly for emergency food alternatives

**DOI:** 10.1038/s41598-023-42140-y

**Published:** 2023-09-13

**Authors:** Diana Nur Afifah, Fitriyono Ayustaningwarno, Anisa Rahmawati, Dhara Nabila Cantikatmaka, Ningsih Wigati, Etika Ratna Noer, Nurmasari Widyastuti, Hartanti Sandi Wijayanti, Denny Nugroho Sugianto, Yesi Pratama Aprilia Ningrum, Vivilia Niken Hastuti

**Affiliations:** 1 Department of Nutrition Science, Faculty of Medicine, Diponegoro University, Semarang, Indonesia; 2 Center of Nutrition Research (CENURE), Diponegoro University, Semarang, Indonesia; 3 SDGs Center, Diponegoro University, Semarang, Indonesia; 4 Department of Oceanography, Faculty of Fisheries and Marine Sciences, Diponegoro University, Semarang, Indonesia

**Keywords:** Biochemistry, Biological techniques

## Abstract

The substitution of wood apple juice and soybean powder in the seaweed jelly product can be used as an alternative to emergency supplementary feeding (ESF) for children under five years of age, which contains high protein, fiber, and calories. This study aimed to determine the effect of adding wood apple juice and soybean powder to the nutrition content, vitamin C, zinc, magnesium, total phenol, antioxidant activity, acceptability, and shelf-life of seaweed jelly products. This study was an experimental study with a completely randomized design with two treatment factors, which consisted of making seaweed jelly products with three different ratios of wood apple juice and soybean powder, 60:40 (F1), 50:50 (F2), and 40:60 (F3), dried at 40 °C (T1) and 50 °C (T2). Macronutrients were determined using proximate analysis. The total phenol and vitamin C were measured using Folin-ciocalteu reagent and UV–Vis spectrophotometry. Antioxidant activity was analyzed by 2,2-diphenyl-1-picryl-hydrazyl-hydrate (DPPH). The contents of zinc and magnesium were evaluated through Atomic Absorption Spectrophotometry (AAS). Estimation of shelf life was determined with Accelerated Shelf-Life Test (ASLT) method and Arrhenius equation model. The best formula based on proximate analysis was F3, which contained 361.98 kcal of energy and 33.79 g of protein. The best formula (F1) dried at 40 °C; contains 56.28 mg/100 g vitamin C; zinc was 1.55 mg/100 g; magnesium was 79.25 mg/100 g; antioxidant activity (IC_50_) was 88.39 μg/mL; and total phenol was 8.59 mg GAE/g. The quality attributes of the best formula show the potential of the jelly as an emergency food despite its short shelf-life.

## Introduction

Indonesia is in a disaster-prone country due to its geographical and geological condition with many records of natural disaster including flooding, droughts, earthquakes, tsunami, storms, tornadoes, landslides and volcanic eruptions^1^. According to the National Board for Disaster Management’s (BNPB), natural disasters across the country recorded more than 3000 incidents in any given year^1^. This situation can decrease food availability due to difficult road access and food sources. The decrease in food availability can also result in nutritional problems, especially for vulnerable groups^2^, including children under five years of age, pregnant women and the elderly^3^. Decreased nutritional status in children often occurs during an emergency including after the disaster and evacuation. During emergency, the number of severely malnourished increased from 1.9% to 5.7%, while moderately malnourished increased from 7.5% to 24.5%^4^.

The supplementary feeding along with main food is recommended for children who are vulnerable to malnutrition in order to fulfill their daily nutritional needs. The supplementary food requirements for undernourished children are 350 kcal with 15 grams of protein^2^. Emergency food is a processed food designed to meet daily needs during an emergency. Emergency food should be safe, acceptable in color, texture, aroma, appearance, nutrition complete, easy to transport, and easy to use. In addition, emergency food products are expected to last a long time; therefore, can be available at any time^5^.

Jelly products, which can be classified into intermediate moisture food (IMF) can be used for emergency food as they have an appealing texture, contribute energy during an emergency, and it has a long shelf life due to the low water content. According to Indonesian National Standard (SNI), the requirement for jelly moisture content is <20%, which will affect the length of shelf life^6^. Furthermore, jelly snack produced with sugar can contribute energy for children’s emergency supplementary feeding. Jelly products is recommended for children, older than two years old who can chew to reduce choking hazard^7^. Local ingredients in Indonesian coastal areas that can be used to produce jelly are *Eucheuma cottonii,* a red seaweed *(Rhodophyceae)* is the most cultivated seaweed in Indonesian costal area. Seaweed contains 12,42% crude fiber^8^ and 2.6% protein which composed by 13 amino acids, with glutamate and aspartate contents as the majority^9^ . Furthermore, *Eucheuma cottonii* contains vitamins, minerals, and phenolic compounds that have antioxidant properties^10^.

Wood apple (*Limonia acidissima* L*.*), known as Kawista (Bahasa) is endemic in Rembang Regency on the north coast of Java, Indonesia, which is used to processed by local into syrup, jam, and *dodol* (a sweet toffee-like sugar palm-based confection). Wood apple contains 2.55 mg/g vitamin C^11^, 9.3 % protein, 3.32% fiber^12^, vitamins B1, B2, phosphorus, magnesium, calcium, iron, and zinc^13^.

Soybeans provide calories and plant-based protein. Compared to other types of legumes soybean contain more protein, vitamins A, B1, and B2^14^. According to the previous research, every 100 grams of soybeans, contains 34.9 g of protein to cover the nutritional needs of supplemental meals for children in an emergency. Soybean has also been shown to be beneficial to human health, which contributed by its bioactive compounds such as isoflavones^15^.

Although many studies on emergency food, they were mostly for adult. Emergency food such as jelly for children was not exist yet. Based on the description above, this research aims were to analyzing nutrition contents, total phenol, antioxidant activity, organoleptic and shelf life of seaweed jelly products with the addition of wood apple and soybeans.

## Methods

### Study design and sample

This is experimental research in a two-factor completely randomized design (CRD). The main ingredients used for making the jelly are seaweed, wood apple and soybean powder. The difference in the ratio of the addition of wood apple juice and soybean powder is based on the results of calculations in preliminary research conducted by researchers with the selected formulation that is most approximate to the requirements of macronutrient content for supplementary food for children under five years in an emergency, which contains energy value of 350 kcal and 15 grams of protein per day^16^. Based on preliminary research ratios of wood apple juice and soybean powder at 60:40 (F1), 50:50 (F2), and 40:60 (F3) was selected by considering the adequacy of total energy and protein in emergency supplementary food for children. The seaweed used is dry *Eucheuma cottonii* as the carrageenan source, purchased from e-commerce, harvested at 45 days, and was planted 25-30 cm below sea level. Wood apple were purcased from Rembang regency through e-commerce. The soybean powder used is the Mandala 525 brand purchased from e-commerce, produced by a single milling process at a low temperature. Gelatin was purchased from a local market in Rembang, while garanulated sugar was produced by PT. Sugar Group Companies, stevia powder by Beorganic, and citric acid by PT. Golden Sinar Sakti. Three formulations containing different proportion of wood apple juice and soybean powder was used, as can be observed in Table 1.

**Tabel 1:** Jelly formulation for 120 g product.

Ingredients	F1	F2	F3
Seaweed (g)	60	60	60
Wood apple (g)	36	30	24
Soy powder (g)	24	30	36
Sugar (g)	15	15	15
Stevia (mg)	50	50	50
Gelatin powder (g)	30	30	30
Citric acid (g)	1	1	1

### Preparation of seaweed jelly production

First, seaweed was soaked in water 1:1 to allow rehydration overnight. Wood apple were pulped and diluted by water to obtain 24% v/v. Then the seaweed and wood apple were homogenized into a paste and filtered using a kitchen sieve. Sugar and stevia then were added and homogenized. Then, to the mixture, dissolved gelatin in 10 ml warm water (±45°C) and soy powder were added. The mixture then homogenized for ±20 minutes at 60–70 °C. Then, the mixture was removed from the heat and citric acid were added. The mixture then was poured into mold and cooled at room temperature and chilled further at 5°C. The jelly then dried for 6 hours at 40 °C (T1) and 50 °C (T2) in a preheated oven^17^.

### Macronutrient analysis

Macronutrient contents were determined using proximate analysis according AOAC 2005^18^. Total energy was calculated using carbohydrate, lipid, and protein content in the product. Fiber content was analyzed using gravimetric method^19^.

### Micronutrient analysis

#### Vitamin C

Vitamin C content was analyzed using UV–Vis spectrophotometer. One hundred mg sample was dissolved in 10 mL of 0.4% oxalic acid, then filtered through filter paper. One ml of the filtrate then diluted using 2,6-dichlorophenol indophenol until 10 ml. The homogenized solution then ready to be measured using UV–Vis spectrophotometer Spectro quant Prove 300 at 561 nm. The standard curve was prepared using pure Vitamin C, at 4, 6, 8, 10 ppm^20^.

### Antioxidant’s activity

Antioxidant activity was analyzed using the DPPH method by calculating the IC_50_ value, which was estimated according to the procedure described by Rustiah^21^. One thousand ppm stock sample solution were produced, filtered, and diluted by methanol to 2, 4, 8, and 16 ppm. An aliquot of 1.5 mL from each concentration then taken and homogenized with 1.5 mL of 40 ppm DPPH in a test tube covered with aluminum foil. The solution then incubated for 30 minutes in dark and ready to be measured using a UV–Vis spectrophotometer Spectro quant Prove 300 at 515 nm^22^. Vitamin C was used as control at 4, 6, 8, 10 ppm.

### Total Phenol

Ten mg of homogenized sample were dissolved with 10 mL of ethanol: distilled water (1:1), then filtered through filter paper. An aliquote of 250 ul filtrate then taken and homogenized with 0.25 mL 95% ethanol; 1.25 mL of distilled water; and 1.25 mL of Folin Ciocalteu reagent (1:10), and then incubated for 5 minutes. After incubated, 0.5 mL of 5% Na_2_CO_3_ solution was added, vortexed, incubated for 1 hour, and the absorbance was measured using a UV–Vis spectrophotometer Spectro quant Prove 300 at 725 nm. Standard solutions of gallic acid at 5, 10, 15, 20, 25, and 50 ppm were used^23^.

### Zinc and magnesium

To prepare a jelly sample, weigh 5 grams of jelly and place it in a ceramic crucible. Five grams of sample were dry ashed at 900 °C in a muffle furnace. After that, the ashes were dissolved in 25 mL of HNO_3_ (1:3). The sample absorbance was measured using an Atomic Absorption Spectrophotometer, Perkin-Elmer 3110 at 213.9 nm for zinc^24^ and 285.2 nm for magnesium^25^.

### Organoleptic Test

Twenty untrained panelists from Diponegoro University's Nutrition Science Department participated in the hedonic analysis. Taste, color, aroma, and texture are all rated using a four-point hedonic scale ranging from 1 (very dislike), 2 (dislike), 3 (likes), and 4 (very like)^26^. This study was approved by the Ethics Committee of the Medical Faculty Sultan Agung Islamic University by ethical clearance *No. 311/IX/2021/Komisi Bioetik,* and all research was performed in accordance with relevant guidelines. Informed consent was obtained from all participants. Ingredients used in the jelly production including seaweed, wood apple, soy powder, sugar, stevia, gelatin powder, and citric acid are safe for human consumption. There were no negative side effects because of this research.

### Total bacteria

Total bacteria was measured using total plate count based on SNI 3547-2-2008^27^.

#### Determination of the best formula

The best formula form three addition ratio of wood apple and soybean flour was chosen based on the multi-attribute decision using compensatory model, additive weighting technique^28^.

#### Shelf-life estimation

The jelly self-life of the best formula was determined using Accelerated Shelf-Life Test (ASLT) method with the Arrhenius equation^29^. To obtain a stable product, the jelly was vacuum packed in polyethylene plastic. The packed jelly then stored for 0, 5, 10, 15, 20, 25, and 30 days at 27 °C, 37 °C, and 47 °C to obtain 21 samples. Each sample was then measured for moisture content by gravimetric^30^, pH, and total bacteria by Total Plate Count (TPC) as of product quality degradation indicators and critical factors that determine shelf life^31–33^.

Deterioration rate of each parameter at each temperature (k) was determined using linier regression equation. Those k values and corresponding 1/T then used to describe the Arrhenius equation in Eq. (1). The k value at expected storage temperature was calculated using Eq. (2). The days of shelf life then estimated using orde 0 as described in Eq. (3).

1 $$\mathrm{ln}k=\mathrm{ln}{k}_{0}-\left(\frac{\mathrm{Ea}}{\mathrm{R}}\right)\frac{1}{\mathrm{T}}$$

2 $$k={k}_{0}.\mathrm{exp}(-Ea/RT)$$

3 $$t=\frac{{A}_{t}-{A}_{0}}{\mathrm{k}}$$

#### Data analysis

Mean values and standard deviation are reported for analytical data. Normality was analyzed using Shapiro–Wilk because the data were less than 30^34^. When the data were normally distributed, One-Way ANOVA analysis was performed for energy, fat, carbohydrates, protein, crude fiber, and moisture content; ash content, zinc, vitamin C, and organoleptic data were analyzed using Kruskal–Wallis test; magnesium, antioxidant activity, and total phenol were analyzed using Two-Way ANOVA. Otherwise, non-normal distributed data were analyzed using the Kruskal–Wallis statistical test. The analysis was continued with the Post-Hoc test when the ANOVA or Kruskal–Wallis test had a significant differences^35^.

#### Ethical approval

The studies involving human participants were reviewed and approved by the Ethics Committee of the Medical Faculty Sultan Agung Islamic University with ethical clearance *No. 311/IX/2021/Komisi Bioetik*. The patients/participants provided their written informed consent to participate in this study.

#### Consent to participate

Participants have given written consent to participate in this study.

#### Consent for publication

Participants agreed to participate in the research study and did not mind having their information published in a journal article.

## Results

### Macronutrient composition

Based on a statistical analysis of the three formulations with One-Way ANOVA, shows that there are significant different variables of energy, fat, carbohydrates, ash content, and crude fiber content with a significance value of *P* ≤ 0.05. Meanwhile, the protein content did not show a significant difference with a significance value of *P* > 0.05. The water content was analyzed using Kruskal Wallis which showed that there was a significant different in the water content variable with a significance value of *P* ≤ 0.05. Table 2 show the results of nutrient contents on seaweed jelly with addition of wood apple and soybean powder.

**Table 2:** Macronutrient composition of seaweed jelly with addition of wood apple and soybean powder per 100 g.

Component	F1	F2	F3	*P*
Energy (kcal)	277.02 ± 22.26^a^	350.52 ± 24.31^b^	402.20 ± 8.61^c^	0.001*
Protein (g)	34.04 ± 2.22	33.08 ± 8.39	37.54 ± 3.73	0.601*
Fat (g)	12.08 ± 1.98^a^	18.85 ± 1.39^b^	27.03 ± 0.91^c^	< 0.001*
Carbohydrates (g)	8.05 ± 3.55^a,b^	12.13 ± 5.45^a^	2.20 ± 1.15^b^	0.050*
Water content (%)	42.25 ± 2.84^a^	33.11 ± 4.86^b^	30.51 ± 2.85^b^	0.017*
Ash Content (%)	3.59 ± 0.34^a^	2.83 ± 0.06^b^	2.73 ± 0.02^c^	0.027**
Crude Fiber (%)	20.08 ± 2.04^a^	22.90 ± 2.16^a,b^	24.97 ± 0.85^b^	0.041*

Seaweed jelly containing wood apple and soybean flour at ratio of 60:40 (F1), 50:50 (F2), 40:60 (F3). Different letter above the standard deviations shows significant different between formulation (*P* < 0.05) by One-Way ANOVA analysis (*) and Kruskal Wallis (**).

### Micronutrient composition

The vitamin C and total phenol content was higher in the jelly dried at 40 °C than 50 °C as can be observed in Table 3. The zinc content in jelly ranges from 1.24 mg/100 g to 1.55 mg/100 g, while the magnesium content ranges from 65.24 mg/100 g to 79.25 mg/100 g. The antioxidant activity of the product varied with the lowest IC_50_ value of 88.39 μg/mL, which was quite strong in the F1 with a drying temperature of 40 °C. However, there were no significant differences in the levels of vitamin C, zinc, magnesium, and the antioxidant activity (*P* > 0.05). The difference in drying temperature of 40 °C and 50 °C significantly influenced the vitamin C and total phenol content. However, it did not affect the content of zinc, magnesium, and the antioxidant activity of the product.

**Table 3:** Micronutrient composition of seaweed jelly with addition of wood apple and soybean powder.

Formulation	Drying temperature (°C)	Vitamin C (mg/100 g) **	Total phenol (mg GAE/g) *	Antioxidant activity (IC_50_ μg/mL) *	Zinc content (mg/100 g) **	Magnesium content (mg/100 g) *
F1	40	56.28 ± 5.44^a^	8.59 ± 0.16^a^	88.39 ± 12.28^a^	1.55 ± 0.42^a^	79.25 ± 12.16^a^
F2	40	55.54 ± 3.97^a^	7.92 ± 0.16^b^	113.62 ± 40.13^a^	1.24 ± 0.02^a^	65.24 ± 4.54^a^
F3	40	54.65 ± 9.75^a^	7.49 ± 0.05^c^	120.51 ± 19.19^a^	1.52 ± 0.66^a^	69.09 ± 11.54^a^
F1	50	49.08 ± 2.81^b^	7.75 ± 0.13^d^	130.94 ± 21.81^a^	1.40 ± 0.13^a^	77.54 ± 3.69^a^
F2	50	47.41 ± 0.42^b^	7.78 ± 0.05^e^	132.22 ± 39.72^a^	1.34 ± 0.13^a^	70.46 ± 7.93^a^
F3	50	46.24 ± 8.20^b^	7.45 ± 0.07^f^	136.38 ± 8.97^a^	1.54 ± 0.09^a^	77.98 ± 3.13^a^

Seaweed jelly containing wood apple and soybean flour at ratio of 60:40 (F1), 50:50 (F2), 40:60 (F3). Different letter above the standard deviations shows significant different between formulation (*P* < 0.05) by Two-Way ANOVA analysis (*), and Kruskal–Wallis analysis (**).

### Organoleptic Test

The formula F1 (60 % wood apple and 40% soybean powder) produced the highest average yield for color, taste, fragrance, and texture due to the addition of wood apple as can be observed in Table 4. Based on the statistical analysis of the three formulations with Kruskal Wallis, it showed that there was no significant difference in the color, aroma and taste variables with a significance value of (*P* > 0.05). When wood apple and soybean powder were combined, the texture variable shows a significant difference with the significance value (*P* < 0.05).

**Table 4:** Hedonic result of jelly with wood apple and soybean powder addition.

Parameter	F1	F2	F3	*P*
Color	3.00 ± 0.70 (like)	2.92 ± 0.75 (like)	3.00 ± 0.57 (like)	0.951**
Aroma	2.72 ± 0.67 (like)	2.56 ± 0.71 (like)	2.60 ± 0.64 (like)	0.642**
Taste	2.76 ± 0.72 (like)	2.40 ± 0.57 (dislike)	2.44 ± 0.71(dislike)	0.128**
Texture	3.00 ± 0.70^a^ (like)	2.24 ± 0.66^b^ (dislike)	2.60 ± 0.57^b,c^ (like)	0.002**

Seaweed jelly containing wood apple and soybean flour at ratio of 60:40 (F1), 50:50 (F2), 40:60 (F3): Different letter above the standard deviations show significant different between formulation (*P* > 0.05) by the Mann–Whitney test, **test with Kruskal Wallis. 1 = very dislike; 2 = dislike; 3 = like; 4 = very like.

### Shelf-Life Estimation

**Figure 1:**
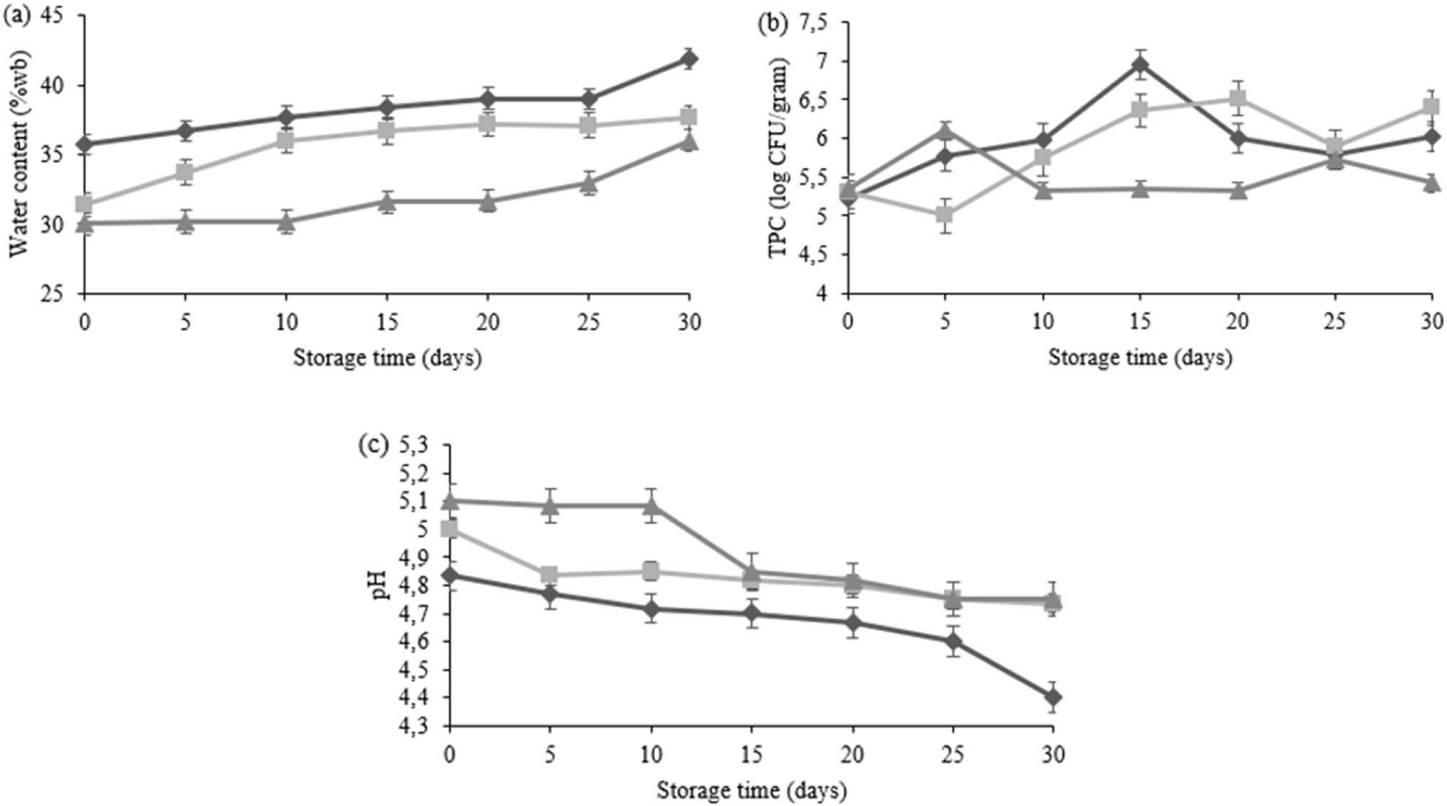
Effect of storage at 27 °C (◆), 37 °C (◼), and 47 °C (▲) on water content (**a**), TPC (**b**), and pH (**c**) of jelly. The error bar is the standard deviation (n = 6).

Figure 1 graphically outlines the parameters used to determine the shelf life with water content, pH, and bacterial count evaluated on days 0, 5, 10, 15, 20, 25, and 30 at 27 °C, 37 °C, and 47 °C. The parameter used to determine the shelf life is the water content with the lowest activation energy value. The k, R2, and Ea values for each parameter can be seen in Table 5. The shelf life of F3 seaweed jelly vacuum packed and stored at 27 °C, 37 °C, and 47 °C was 48.2 days, 47.7 days, and 47.2 days, respectively, as presented in Table 6.

**Table 5:** Kinetic parameters of the shelf-life estimation for each quality attribute.

Parameter	Temp. (^o^C)	k	R^2^	Ea (kcal/mol)
Water content	27	0.1749	0.4202	0.2165
	37	0.1922	0.6415	
	47	0.1786	0.2984	
pH	27	− 0.0120	0.7888	0.8466
	37	− 0.0073	0.5020	
	47	− 0.0142	0.7771	
Bacterial Count	27	0.0173	0.0420	− 12.1411
	37	0.0415	0.3852	
	47	− 0.0033	0.0032

**Table 6:** Shelf life of seaweed jelly.

Temperature	Initial Water Content	Final Water Content^36^	k	Shelf Life (days)	Shelf Life (months)
27	31.33	40	0.180	48.2	1.61
37	31.33	40	0.182	47.7	1.59
47	31.33	40	0.184	47.2	1.57

### Determination of the best formula

The best formula of seaweed jelly with wood apple and soybean powder addition are presented in Table 7.

**Table 7:** The best formulation based on multi-attribute decision using compensatory model, additive weighting technique.

Formula/Result Value (Nh)	F1	F2	F3
Energy	0.00	0.11	0.19
Protein	0.04	0.00	0.17
Organoleptic	0.15	0.02	0.00
Fat	0.13	0.07	0.00
Carbohydrate	0.07	0.12	0.00
Crude fiber	0.00	0.06	0.19
Water content	0.00	0.06	0.08
Ash content	0.00	0.05	0.06
Total Yield Value (Nh)	0.39	0.44	0.60

Seaweed jelly containing wood apple and soybean flour at ratio of 60:40 (F1), 50:50 (F2), 40:60 (F3). Nh is obtained from multi-attribute decision using compensatory model, additive weighting technique.

Furthermore, the recommended jelly product based on the highest content of vitamin C, zinc, magnesium, and antioxidants is the first formulation with a drying temperature of 40 °C can be seen in Table 8.

**Table 8:** Quality attribute of jelly.

Micronutrients and Antioxidants	Contents Per 127 Grams	RDA^37^	
		2–3 y.o	4–6 y.o
Vitamin C (mg)	71.48	40	45
Zinc (mg)	1.97	3	5
Magnesium (mg)	100.65	65	95
Total phenol (mg GAE/g)	8.59	–	–
Antioxidant activity (IC_50_ μg/mL)	88.39 (strong)	–	–

## Discussion

Energy, protein, fat, and crude fiber of the jelly product were increased as the portion of wood apple decreased, and soy powder increased. This increase was caused by soybean powder as one of the main ingredients, has higher energy, protein, fat, and crude fiber content than wood apple. The energies of soy powders per 100 g was 450 kcal, containing 38.8% protein, 71.6% fat, and 11.3% fiber^38^. Meanwhile, wood apple contains 120 kcal/100 g, 3.5% protein, 2.5% fat, and 4.6% fiber^39^.

Previous studies have discussed the effect of adding soy flour to increase product fat content. Soy flour addition increased fat content from 17.36% to 20.89% in soy-mushroom-enriched biscuits with soy flour supplementation compared to control biscuits without soy flour addition^40^. However, high fat content in the soy flour is susceptible to the lipoxygenase-catalyzed oxidation of unsaturated fatty acids, which leads to a deterioration of flavor and a reduction in the shelf-life of food products^41^.

Carbohydrates, water, and ash content of the jelly product were decreased as the portion of wood apple decreased, and soy powder increased. Similar trend in decrease in moisture and ash content was also reported by Farzana and Mohajan^40^ on the supplementation of soy flour on the production of soy-mushroom-enriched biscuits. The decrease in carbohydrate could be caused by the addition soy flour which has been low carbohydrate content are in agreement with study of Ayo, et al.^42^, while similar results also showed that the water and ash content gradually decreased from 42.25–30.51% to 3.59–2.73% with the increase in soy flour contained more total dry solids with high emulsifying properties than wood apple^40^. Water content also decreased because wood apple was mostly moisture and soy flour were contained very low moisture.

Based on Indonesian National Standard 2008, jelly should have moisture content less than 20%, while the ash content should be less than 3%^6^. However, the water content of the three formulations did not fulfill the specified Indonesian National Standard requirements. This can be due to the low amount of sugar used, while the addition of high amount of sugar can reduce the water content. The high concentration of sugar can lead to osmotic dehydration, which resulting in the release of some water from the material^43^. Meanwhile, the ash content in F2 and F3 fulfilled the Indonesian National Standard requirements for ash content in jelly, while F1 did not fulfill the requirements^6^.

The vitamin C content was higher in the jelly dried at 40 °C than 50 °C. This result is consistent with research which previously preparing pineapple jam boiled at 90 °C and 105 °C. This indicates that the highest and lowest vitamin C content was in pineapple jam boiled at 90 °C (9.90 mg/100 g) and 105 °C (7.74 mg/100 g)^44^. The reduction in vitamin C is related to an increase in drying temperature, as vitamin C is easily degraded during the heating process. Therefore, the vitamin C is sensitive to heat, light, and high temperature during processing^44^. Polka, et al.^45^ showed that hot-air/oven drying of fruits reduced vitamin C content by 30–72 % and carotenoids by 0–90 %. The mechanism of decreasing content in food products occurs due to the oxidation of vitamin C. Vitamin C is easily oxidized in the presence of oxygen, light and heavy metal ions^46^.

Zinc and magnesium content of seaweed jelly were decreasing as drying temperature was increased. According to previous research, the zinc content of boiled cowpeas (1.44 mg/100 g) was lower than fresh cowpeas (3.5 mg/100 g) due to heating, exposure to oxygen, sunlight or its combination^47^. Meanwhile, the magnesium content of roasted spinach (411 mg/100 g)^48^ was also lower than fresh spinach (430 mg/100 g).

IC_50_ of jelly dried at 40 °C was lower compared to jelly dried at 50 °C. A lower IC_50_ shows a higher antioxidant activity. The antioxidant activity of the jelly was contributed by the phenolic compounds and vitamin C which usually present in fruit and vegetable in a high concentration. However, phenolic compounds and vitamin C are thermal sensitive compounds. Phenolic compounds and vitamin C degradation can reduce the antioxidant activity. Vitamin C and phenolic compounds are important in the diet because of their function as antioxidants and a defense mechanism to neutralize reactive oxygen species (ROS) to prevent molecular damage^43,49,50^.

Phenols are important ingredients for antioxidant-rich foods^51^. F1 formulation jelly contained the highest total phenol, followed by the second and third. This is related to the difference in the ratio of wood apple extract and soybean flour added to each formulation. More wood apple applied to the jelly resulted in a greater overall phenol concentration. This is because wood apple as the main ingredient for making jelly, is rich in phenolic compounds. The total phenol content of this fruit is 0.75-22 mg GAE/g^52^. Additionally, *Eucheuma cottoni* seaweed also contributed the total phenol content with 141 mg GAE/g^53^.

Formula F1 (60% wood apple and 40% soybean powder) showed the highest hedonic score for color, taste, aroma, and texture due to the high protion of wood apple. Wood apple has a distinctive sweet and sour taste^54^, and the panelists preferred the strong aroma of wood apple^54^. Texture of F1 was the highest among other formulas, wood apple contain pectin^55^, that have a high water binding capacity^30^ which promoting chewy texture, and liked by consumer.

Consumer liked all formula jelly color. However, jelly color was different for F1 which have higher wood apple content and F3 which have higher soy powder content. Wood apple has a dark brown color, making the product dominantly brown. Meanwhile, the addition of soybean powder to F3 caused the protein content to increase. The higher the product’s protein content, the darker the color produced due to the Maillard reaction^56^. Furthermore, F1 and F3 did not have a significant (*P* > 0.05) color difference because both formulations were dark brown due to the dominant color of higher wood apple in F1 (60%) and higher soy powder in F3 (60%).

Shelf life was estimated using the quality attribute with lowest activation energy or the most sensitive parameters to temperature changes which is water content^57^. Figure 1a showed that the lowest water content was observed in jelly stored at 47 °C. Products stored at higher temperatures produce lower water content because the storage temperature is directly proportional to the evaporation process also as reported by Tobal & Rodrigues^49^. Vacuum can also reduce the water content during storage because all initial water vapor and air have been sucked out of the packaging. As a result, it becomes airtight and prevents water from penetrating the product^58^ However, during storage moisture was released from the jelly matrix as syneresis indicator . Seaweed is source of carrageenan, which is a hydrocolloid, able to maintain moisture content and protecting the gel from syneresis^59^.

Initial pH of jelly was influenced by the product composition including wood apple and citric acid concentration. Wood apple juice had a pH of 3.2 and decreasing jelly pH with the increase of wood apple concentration. Citric acid was added to decreasing pH of seaweed jelly as well to inhibit microbial growth^60^. Figure 1c showed pH of jelly stored at all temperatures were decreased gradually with the highest decrease was jelly stored 27 °C. pH changes were related to the water content. The increase of water content was followed by the decrease in pH. The onset of syneresis would be related to decrease of pH. A previous study stated that syneresis can increase at lower pH values due to the acidic environment modifying the chemical structure of the jelly after several days of storing^61^.

Total plate count of the jelly was increased and then decreased following the bacteria growth phases. Figure 1b showed that on days 0-5, bacterial growth at 27 °C and 37 °C were in a lag phase in which they were still adapting to the new environment. Furthermore, on day 5-15 and 5-20, at 27 °C and 37 °C, the growth experienced an exponential phase characterized by bacteria growing at a logarithmic speed. Moreover, on day 20-25 at 27 °C and 37 °C, there was a stationary phase with a decrease in growth rate due to lack of nutrients, changes in pH, or other factors. The last is the death phase, where more dead cells are more than the living cells.

Jelly stored at 27 °C had a shelf life of 1.61 months. Emergency food should have a shelf life of 36 months at 21 °C storage^62^. In order to increase the shelf life, hydrocolloid used should be decreased to 7.3%, and sugar should be increased to 55%; and keep the water content about 14.32%^63^. The concentration of hydrocolloid material is directly related to the water content. This is because water can be bound by hydrocolloids in the gel formation process, and the lower water content can result in a longer shelf life^64^.

F3 was the best jelly with portion of wood apple and soybean powder of 40:60 based on macronutrients, including moisture, ash, crude fiber content, and organoleptic. F3 has fulfilled 361.98 kcal of energy and 33.79 grams of protein per day as required by the ESF standard. Emergency supplementary feeding standard is 350 kcal of energy and 15 grams of protein per day, which means about 30 pcs/day or 10 pcs/serving can be consumed. The high protein content in the product is expected to help children during an emergency. Low protein intake can affect the immunity, which can cause infectious diseases and reduce nutritional status. The quality attributes of the best formulation show the potential of the jelly as emergency food despite the short shelf-life.

## Conclusion

Seaweed jelly with wood apple fruit and soybean powder can be used as an alternative to ESF for children. The best formulation based on macronutrients, moisture content, ash content, crude fiber, and organoleptic is F3 with energy and protein capable of fulfilling daily needs of 361.98 kcal and 33.79 grams of protein per day. The shelf life of seaweed jelly with the addition of wood apple extract and soybean powder vacuumed and stored at 27°C, 37°C, and 47°C, was 48.2 days, 47.7 days, and 47.2 days. The quality attributes of the best formulation show the potential of the jelly as emergency food despite the short shelf-life, allowing for future development to include ingredient and composition modification to increase the shelf life.

### Supplementary Information

Supplementary Figure S1.

Supplementary Table S1.

## Data Availability

The study's original contributions are included in the article/supplementary material; further questions can be directed to the corresponding authors.
