# Supplementary material for: Characteristics of wood apple (Limonia acidissima L.) and soybean powder jelly for emergency food alternatives
Source: Sci Rep. 2023 Sep 13;13:15161. doi: 10.1038/s41598-023-42140-y (PMC10499880; doi:10.1038/s41598-023-42140-y)

Supplementary Figure

Characteristics of Wood Apple (Limonia acidissima L.) and Soybean Powder Jelly for Emergency Food Alternatives

Diana Nur Afifah, Fitriyono Ayustaningwarno, Anisa Rahmawati, Dhara Nabila Cantikatmaka, Ningsih Wigati, Etika Ratna Noer, Nurmasari Widyastuti, Hartanti Sandi Wijayanti, Denny Nugroho Sugianto, Yesi Pratama Aprilia Ningrum, Vivilia Niken Hastuti

Figure S1. Photograph of wood apple and soybean powder jelly with different formulation


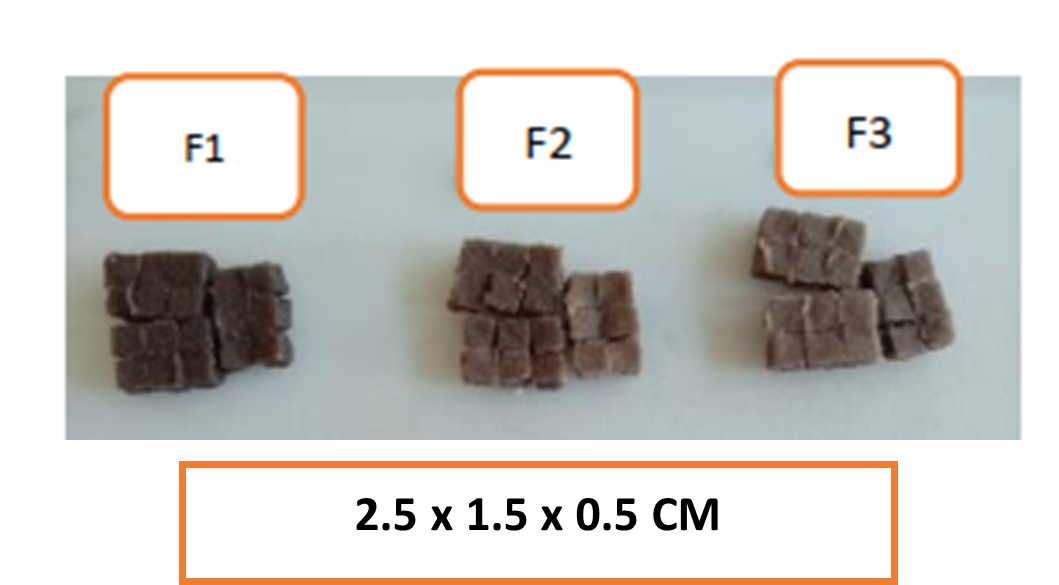

Supplement: Supplementary file 1 — Supplementary Figure S1. [file 41598_2023_42140_MOESM1_ESM.docx]
