# Supplementary material for: Characteristics of wood apple (Limonia acidissima L.) and soybean powder jelly for emergency food alternatives
Source: Sci Rep. 2023 Sep 13;13:15161. doi: 10.1038/s41598-023-42140-y (PMC10499880; doi:10.1038/s41598-023-42140-y)
Supplement: Supplementary file 2 — Supplementary Table S1. [file 41598_2023_42140_MOESM2_ESM.docx]

Supplementary Table

Characteristics of Wood Apple (Limonia acidissima L.) and Soybean Powder Jelly for Emergency Food Alternatives

Diana Nur Afifah, Fitriyono Ayustaningwarno, Anisa Rahmawati, Dhara Nabila Cantikatmaka, Ningsih Wigati, Etika Ratna Noer, Nurmasari Widyastuti, Hartanti Sandi Wijayanti, Denny Nugroho Sugianto, Yesi Pratama Aprilia Ningrum, Vivilia Niken Hastuti

Table S1. List of abbreviations

| A_0_ | initial quality value |
| --- | --- |
| AAS | atomic absorption spectrophotometry |
| AOAC | association of official analytical chemists |
| ASLT | accelerated shelf-life test |
| A_t_ | value of product quality remaining after time t |
| CRD | completely randomized design |
| DPPH | 2,2-diphenyl-1-picryl-hydrazyl-hydrate |
| Ea | activation energy (J mol^-1^) |
| ESF | emergency supplementary feeding |
| IMF | intermediate moisture food |
| k | rate constant of the reaction (day^-1^) |
| k_0_ | Arrhenius constant |
| R | gas constant (1.986 cal mol^-1^ K^-1^ or 8.314 J mol^-1^K^-1^) |
| T | absolute reaction temperature (K) |
| t | prediction of shelf life (days) |
| TPC | total plate count |
